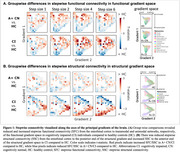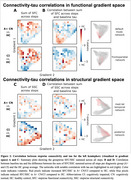# Mapping tau spread to long‐range functional and structural connections along the major axes of brain organization

**DOI:** 10.1002/alz70862_109745

**Published:** 2025-12-23

**Authors:** Jazlynn Xiu Min Tan, Min Su Kang, Yi‐Hsuan Yeh, Nesrine Rahmouni, Gleb Bezgin, Firoza Z Lussier, Seok Jun Hong, Jonah Isen, JoAnne McLaurin, Boris Bernhardt, Bojana Stefanovic, Jean‐Paul Soucy, Serge Gauthier, Sandra E. Black, Pedro Rosa‐Neto, Maged Goubran, Julie Ottoy

**Affiliations:** ^1^ Department of Medical Biophysics, University of Toronto, Toronto, ON Canada; ^2^ Dr. Sandra E. Black Centre for Brain Resilience and Recovery, LC Campbell Cognitive Neurology, Hurvitz Brain Sciences Program, Sunnybrook Research Institute, University of Toronto, Toronto, ON Canada; ^3^ Translational Neuroimaging Laboratory, The McGill University Research Centre for Studies in Aging, Montréal, QC Canada; ^4^ University of Pittsburgh, Pittsburgh, PA USA; ^5^ Center for Neuroscience Imaging Research, Institute for Basic Science, Suwon, Suwon Korea, Republic of (South); ^6^ Biological Sciences, Sunnybrook Research Institute, Toronto, ON Canada; ^7^ McConnell Brain Imaging Centre, Montreal Neurological Institute and Hospital, McGill University, Montreal, QC Canada; ^8^ Sunnybrook Research Institute, Toronto, ON Canada; ^9^ Montreal Neurological Institute, McGill University, Montréal, QC Canada; ^10^ Dr. Sandra E. Black Centre for Brain Resilience and Recovery, Toronto, ON Canada

## Abstract

**Background:**

Prior studies have focused on tau spread from the entorhinal cortex along first‐order (short‐range) connections within anatomical brain space (seed‐to‐target). Here, we explore tau spread along long‐range connections within a novel coordinate space called connectome gradients, which is reflective of the brain’s hierarchical organization. Exploring tau spread within this new framework offers insights into long‐range connectivity alterations and network susceptibility in Alzheimer’s disease (AD).

**Method:**

We included 213 participants from TRIAD (103 A‐ CN, 103 A+ CN, and 75 A+ CI) with diffusion‐weighted MRI, resting‐state functional MRI, and 18F‐MK6240 tau‐PET. First, we employed graph theory‐based stepwise connectivity analyses to unveil long‐range connectivity patterns from the entorhinal cortex to the rest of the brain. Differences in connectivity patterns were compared between A+ vs A‐ groups, adjusted for age, sex, and APOE‐ε4. Second, we investigated the stepwise connectivity patterns in relation to tau within a novel coordinate system spanned by the principal functional and structural connectome gradients.

**Result:**

In the preclinical stage (A+ CN) compared to controls (A‐ CN), we observed connectivity increase through functional gradient space (Figure 1A red). In the clinical stage (A+ CI), connectivity reduced from the entorhinal cortex to the transmodal end of the functional gradient (DMN/limbic; Figure 1A blue) and to the posterior end of the structural gradient (temporo‐occipital; Figure 1B blue). Long‐range connections from the entorhinal cortex showed increased connectivity toward the unimodal and anterior ends of the functional and structural gradient, respectively (Figure 1A, B red), potentially initiating new paths for tau spread. Indeed, tau–connectivity correlations shifted spatially within gradient space with disease progression (Figure 2), moving from the highest‐order (DMN/limbic) cognitive system of the functional gradient in A+ CN to the second‐highest order (frontoparietal) system in A+ CI.

**Conclusion:**

We employed a novel integration of stepwise connectivity and connectome gradients to enable a better understanding of how connectivity is related to tau spread along the major axes of brain organization. We observed widespread network reorganization in AD and notably that the tau–connectivity correlations shifted between major networks of the functional connectome gradient with disease progression.